# Focused scores enable reliable discrimination of small differences in steatosis

**DOI:** 10.1186/s13000-018-0753-5

**Published:** 2018-09-20

**Authors:** André Homeyer, Seddik Hammad, Lars Ole Schwen, Uta Dahmen, Henning Höfener, Yan Gao, Steven Dooley, Andrea Schenk

**Affiliations:** 1 Fraunhofer MEVIS, Am Fallturm 1, 28359 Bremen, Germany; 2 Section Molecular Hepatology, Department of Medicine II, Medical Faculty Mannheim, Heidelberg University, 68167 Mannheim, Germany; 3 Department of Forensic Medicine and Toxicology, Faculty of Veterinary Medicine, South Valley University, Qena, 83523 Egypt; 4 Department of General, Visceral and Vascular Surgery, Jena University Hospital, Drackendorfer Str. 1, 07747 Jena, Germany

**Keywords:** Automated image analysis, Fatty liver, Heterogeneity, Histology, Hotspot analysis, Steatosis, Tile-based analysis

## Abstract

**Background:**

Automated image analysis enables quantitative measurement of steatosis in histological images. However, spatial heterogeneity of steatosis can make quantitative steatosis scores unreliable. To improve the reliability, we have developed novel scores that are “focused” on steatotic tissue areas.

**Methods:**

Focused scores use concepts of tile-based hotspot analysis in order to compute statistics about steatotic tissue areas in an objective way. We evaluated focused scores on three data sets of images of rodent liver sections exhibiting different amounts of dietary-induced steatosis. The same evaluation was conducted with the standard steatosis score computed by most image analysis methods.

**Results:**

The standard score reliably discriminated large differences in steatosis (intraclass correlation coefficient ICC = 0.86), but failed to discriminate small (ICC = 0.54) and very small (ICC = 0.14) differences. With an appropriate tile size, mean-based focused scores reliably discriminated large (ICC = 0.92), small (ICC = 0.86) and very small (ICC = 0.83) differences. Focused scores based on high percentiles showed promise in further improving the discrimination of very small differences (ICC = 0.93).

**Conclusions:**

Focused scores enable reliable discrimination of small differences in steatosis in histological images. They are conceptually simple and straightforward to use in research studies.

## Background

Hepatic steatosis describes the pathological accumulation of fat in the liver. It is the defining characteristic of fatty liver disease (FLD), one of the most common liver disorders in the Western world [1]. Without treatment, FLD can progress into steatohepatitis, cirrhosis, and hepatocellular carcinoma [2]. In clinical routine, steatosis is assessed to determine the severity of FLD or the selection of grafts suitable for liver transplantation. In research studies, steatosis is assessed in order to investigate risk factors of FLD, like alcohol abuse, obesity, or drug toxicity, and to develop anti-steatotic therapies [2, 3].

**Fig. 1:**
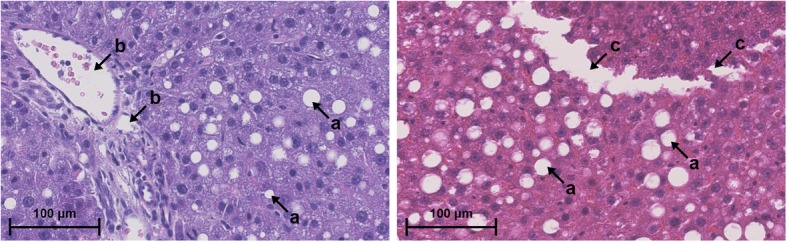
Appearance of steatosis. In histological sections, macrovesicular steatosis appears as white fat droplets (**a**) in the cytoplasm of hepatocytes. They must be distinguished from other white structures like vessels (**b**) or tissue cracks (**c**)

Histological analysis is the gold standard for assessment of steatosis [4]. For this purpose, liver tissue samples are processed into paraffin-embedded slides and stained with Hematoxylin and Eosin (H&E). This paper exclusively considers macrovesicular steatosis, which is commonly assessed in clinical routine and research studies [5, 6]. Under the microscope, macrovesicular steatosis appears as white roundish fat droplets in the cytoplasm of hepatocytes. These fat droplets must be distinguished from other white structures, such as vessels or tissue cracks (see Fig. 1).

**Fig. 2:**
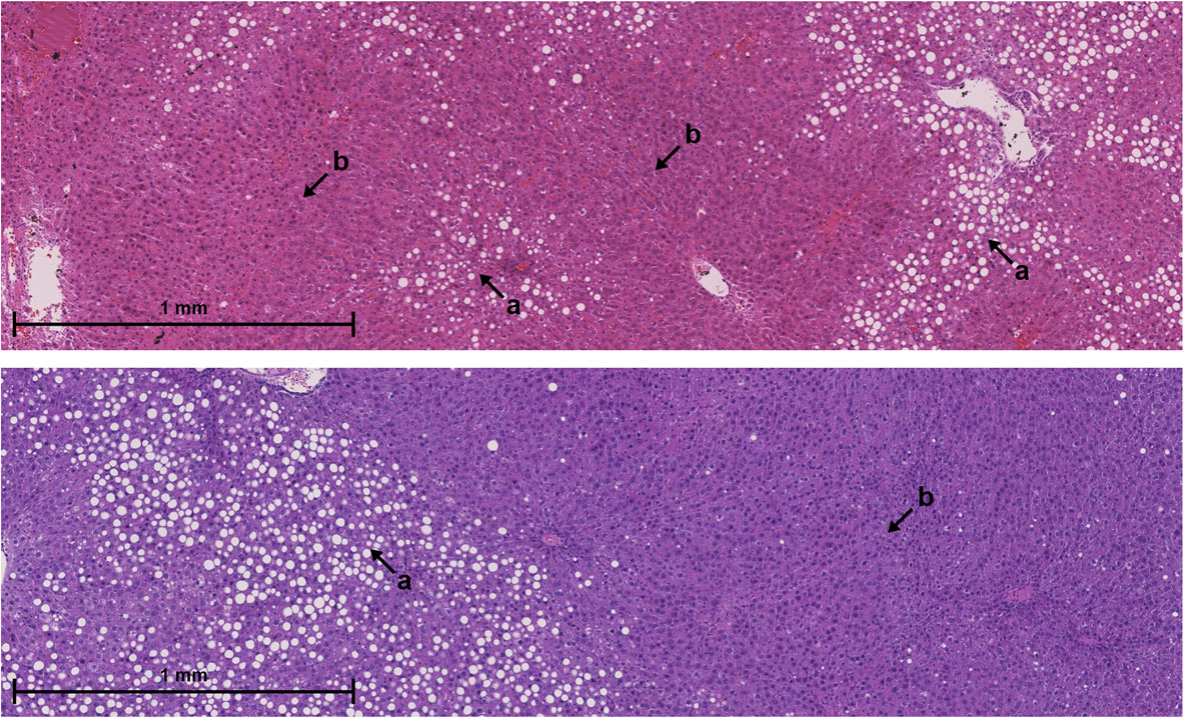
Spatial heterogeneity of steatosis. Steatotic areas (**a**) and non-steatotic areas (**b**) are distributed heterogeneously across the tissue

Steatosis is typically distributed heterogeneously across the tissue. In steatotic areas, hepatocytes contain fat droplets of different numbers and sizes. In non-steatotic areas, hepatocytes do not contain any fat droplets (see Fig. 2). It is common to distinguish between diffuse and focal distributions. Diffuse steatosis often reflects the lobular structure of the liver and is concentrated near portal fields or central veins [7]. Focal steatosis is confined to clearly defined regions surrounded by large areas of non-steatotic tissue [8].

Visual estimation by a hepatopathologist is the traditional method of assessing steatosis in histological slides [4]. However, in recent years, various automated analysis methods were developed to make the measurement of steatosis in histological images more efficient and reproducible [9–14]. These methods quantify steatosis in terms of the steatosis area fraction, that is, the area fraction of macrovesicular fat droplets with respect to the total tissue area.

Spatial heterogeneity of steatosis can make the quantification of steatosis area fractions unreliable. For one thing, steatosis area fractions are sensitive to the proportion of steatotic to non-steatotic tissue in the sample, which is difficult to standardize. For another thing, if non-steatotic areas are much larger than steatotic areas, then steatosis area fractions are often so small that they become sensitive to minor image analysis errors.

Hotspot analysis is a common approach to quantify heterogeneously distributed tissue parameters. The idea is to consider only regions with particularly high or abnormal values which are assumed to be characteristic for the parameter distribution [15]. Hotspot analysis is routinely performed in the assessment of the Ki67 proliferation index [16] and other quantitative tissue parameters, like PD-L1 biomarker expression [17], or tumor vascularity [18]. Its results critically depend on both the location and the size of the considered regions [16]. When performed manually, the selection of both the location and the size of hotspot regions tends to be very subjective [15, 18, 19].

Tile-based approaches make hotspot analysis more objective. They divide tissue images into a regular grid of tiles and determine tissue parameters for each tile individually. Both operations are performed by automated image analysis. This makes it possible to objectively select hotspot regions based on the parameter values of tiles. Furthermore, it enables characterization of the spatial distribution of a tissue parameter through statistics about its values across the tiles.

Plancoulaine et al. describe a tile-based approach to objectify the hotspot analysis of the Ki67 proliferation index [19]. First, they select a stable proportion of tiles with high proliferation values, and then they compute the Ki67 proliferation index as a percentile of the values of the selected tiles. Nawaz et al. describe an approach for the development of novel prognostic scores for estrogen-receptor-negative breast cancer [20]. They select clusters of tiles containing high numbers of tumor or immune cells, respectively, and subsequently they compute statistics about the co-localization of the selected tiles.

In this paper, we present novel scores for reliable quantification of heterogeneously distributed steatosis. The idea is to focus the computation of scores on steatotic tissue areas in order to reduce their sensitivity to tissue sampling and to image analysis errors. Hence, the scores were named “focused scores”. For the computation of focused scores, we adopted concepts of tile-based hotspot analysis in order to select steatotic areas in an objective way. We evaluated different variants of focused scores in terms of their ability to discriminate differences in steatosis. For comparison, the same evaluation was conducted with the standard score computed by most image analysis methods.

## Methods

### Score computation

**Fig. 3:**
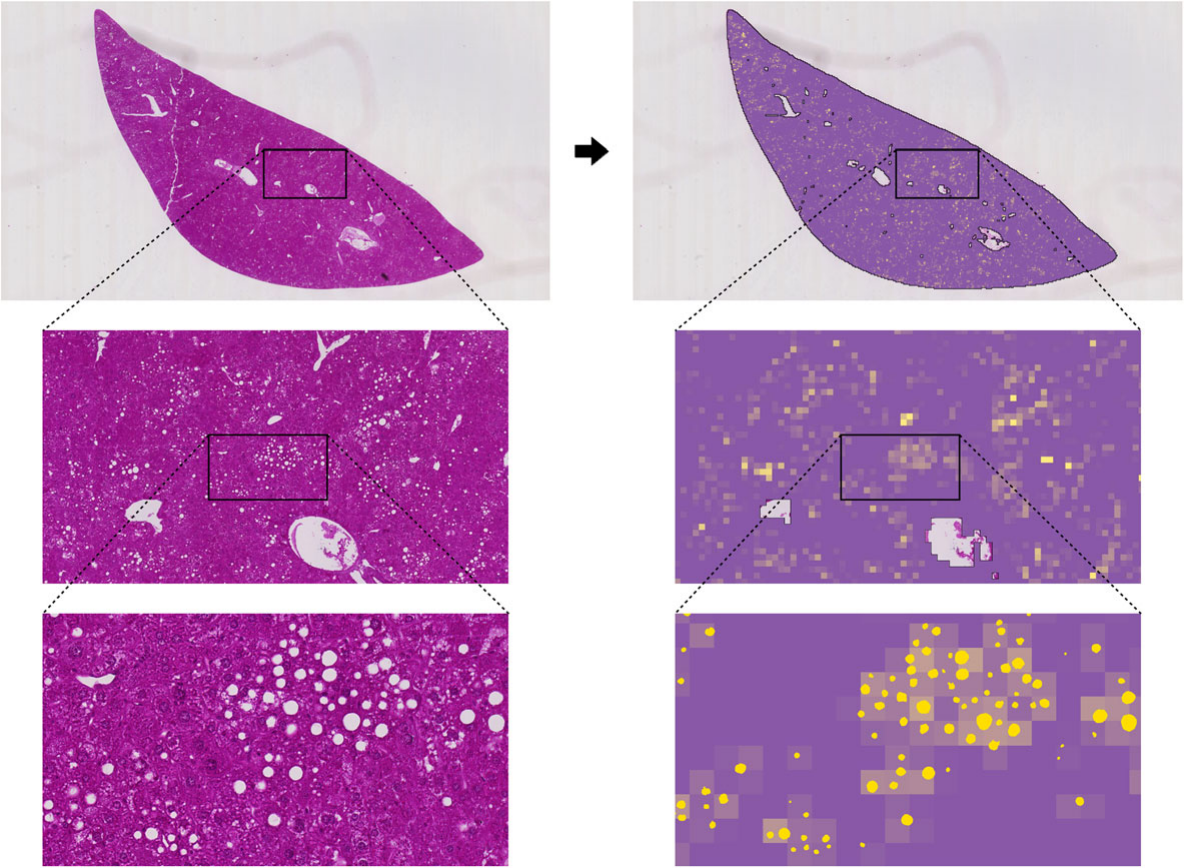
Tile-based steatosis quantification. The steatosis area fractions of tiles are visualized as colors from purple to yellow. At highest magnification, the identified fat droplets are masked in yellow

**Fig. 4:**
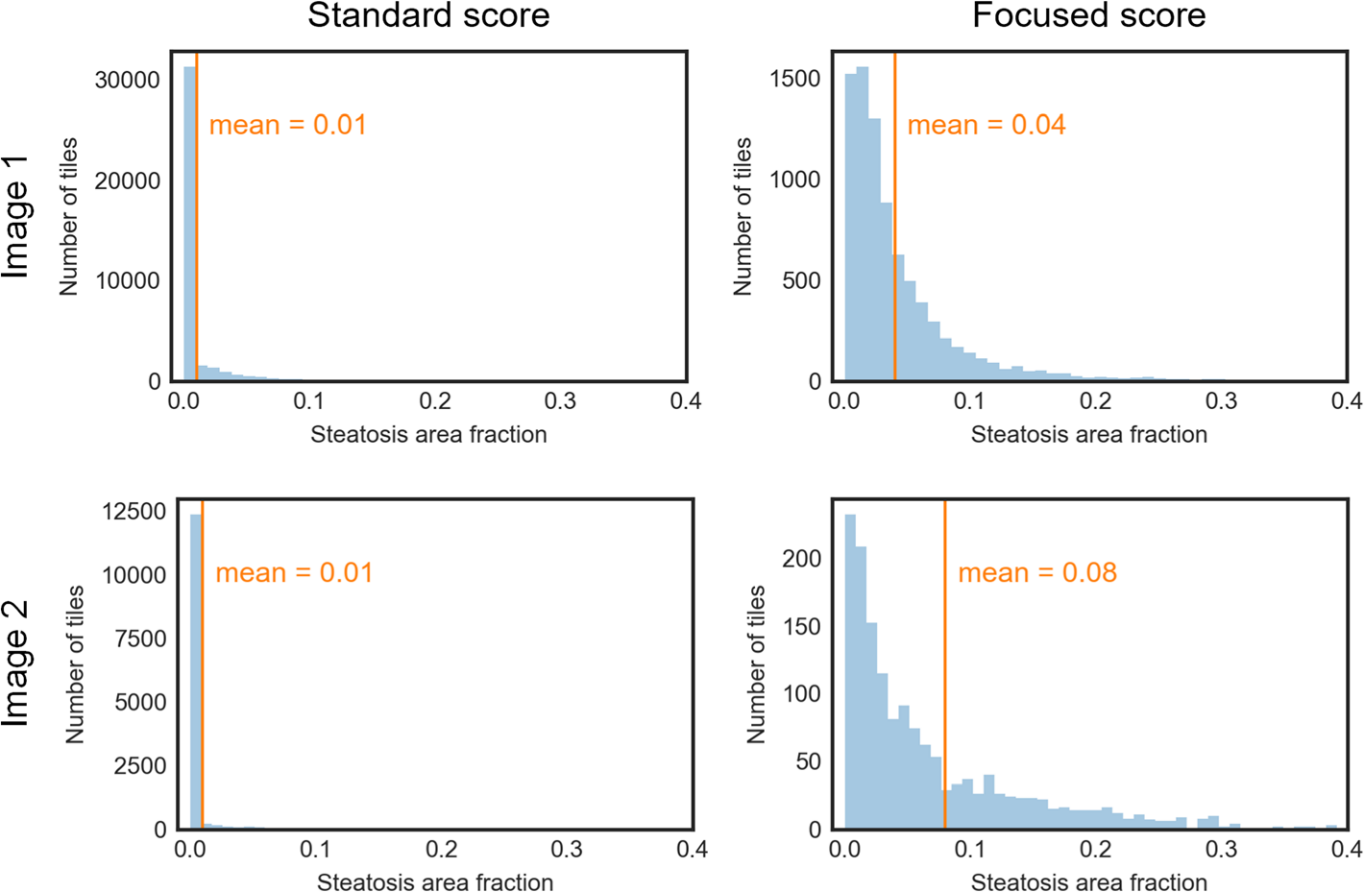
Score computation. The plots show distributions of tile-based steatotic area fractions of two example images. The columns illustrate the computation of the standard score and a mean-based focused score, respectively. While all tiles are considered for the standard score, only steatotic tiles are considered for the focused score. The large peak at value 0 makes the standard scores of both images indistinguishable

All steatosis scores considered in this paper were computed in a simple two-step process. First, a whole-slide image of a H&E-stained liver section was divided into a grid of square tiles and the steatosis area fractions in the individual tiles were determined by automated image analysis (see Fig. 3). Second, the score value was computed as a summary statistic about the steatosis area fractions of selected tiles (see Fig. 4).

This process is independent of the particular method for computing steatosis area fractions and arbitrary methods can be used. In [9], different methods for computing steatosis area fractions were compared in terms of their agreement with human observers. One algorithm was significantly superior to the others and suggested as a suitable automated replacement for manual analysis. We created a custom software implementation of this algorithm and used it for the evaluation of steatosis scores.

The algorithm for computing steatosis area fractions was applied at an image resolution of 454 nm/pixel (approx. 20× magnification). First, pixels were classified as foreground or background. Afterwards, blobs of connected foreground pixels were classified as fat droplets or other white structures, such as vessels or tissue cracks. Both operations were performed with machine-learning classifiers, using features derived from saturation and brightness values for the pixel classification, and shape features for the blob classification.

#### Standard score

The steatosis score computed by most automated image analysis methods is the steatosis area fraction within the entire section. We used this “standard score” as the baseline for the evaluation of novel steatosis scores in this paper.

When sections are divided into tiles of the same size, the standard score roughly equals the mean of the steatosis area fractions of all tiles. As long as tiles are small enough to accurately cover the entire tissue section, the standard score is practically unaffected by the tile size. For this reason, we computed the standard score with a small tile size of 8 μm.

#### Focused scores

In addition to the standard score derived from all tiles, we considered focused scores that were only derived from steatotic tiles. Steatotic tiles were required to have a steatosis area fraction greater than zero. A minimum of 100 steatotic tiles were required in order to obtain a sound estimate of the statistic, or else the score was deemed undefined.

In contrast to the standard score, focused scores are affected by the tile size. The tile size determines the level of detail with which steatotic areas are selected. We considered focused scores based on tiles with edge lengths of 8 μm, 16 μm, 32 μm, 64 μm, or 128 μm, respectively. The tile sizes cover the size range from small macrovesicular fat droplets (8 μm) to groups of 10–20 hepatocytes (128 μm).

Furthermore, we considered focused scores based on the mean and on certain percentiles of the steatosis area fractions of steatotic tiles. The mean is a reasonable statistic for characterizing symmetric and mound-shaped distributions, such as the normal distribution. Tile-based distributions of steatosis area fractions, however, tend to be asymmetric and heavily skewed to the right (see Fig. 4). This applies even when considering only steatotic tiles.

For asymmetric and skewed distributions, percentiles are more appropriate statistics because they make no assumptions about the shape and are robust towards outliers. Percentiles are simple to compute and interpret. The 50th percentile, also called median, reflects the central tendency of a data set. Low and high percentiles, such as the 10th or 90th percentiles, reflect its spread to the left or right. We considered focused scores based on the 10th, 20th, 30th, 40th, 50th, 60th, 70th, 80th, and 90th percentile.

### Data sets

We evaluated the scores on three preexisting data sets of images of H&E-stained rodent liver sections, denoted as data set A, B, and C. Every data set was divided into multiple groups of images, with each group representing a distinct level of steatosis. Steatosis scores were expected to vary significantly between groups but insignificantly within groups. Also, inter-group differences were expected to be large in data set A, small in data set B and very small in data set C.

#### Data set A

Data set A contained 24 whole-slide images of rat liver sections. The data set was divided into four groups of six male Lewis rats each. The individual groups were fed different diets (Ssniff Spezialdiäten GmbH, Soest, Germany) for 3 months:

- Ctrl: Normal rat chow
- D1: Low methionine-low choline plus high starch diet
- D2: Low methionine-low choline plus high fat diet
- D3: Methionine-choline-deficient diet

At the end of the feeding periods, a 70% partial hepatectomy was performed and liver tissue was collected for further analysis. Sections of the left lateral lobe and the median lobe were stained with H&E and scanned with a Hamamatsu NanoZoomer HT 2.0 whole-slide scanner at a resolution of 227 nm/pixel.

Steatosis scores were expected to vary significantly between groups because the diets differed in their capacity to induce steatosis. However, only insignificant differences were expected within groups because the respective animals were of the same strain and fed with the same diet for the same time. The groups were sorted according to the average steatosis level induced by the respective diet, as determined by the mean steatosis area fraction within sections.

Rats were obtained from the Central Animal Laboratory, University Hospital Essen, Germany. All procedures were carried out in accordance with German animal welfare legislation.

#### Data set B

Data set B contained 30 whole-slide images of sections of murine left liver lobes. The data set was divided into five groups of six mice each. All animals were male C57BL/6J mice that were treated according to the STAM model [21] for variable periods of time. The severity of steatosis in this model is related to the feeding time and includes the whole disease spectrum of FLD including hepatocellular carcinoma.

On the second day after birth, the mice were given a single subcutaneous injection of 200 μg streptozotocin (Sigma, MO, USA) to induce insulin deficiency and produce a model of diabetes (first hit). Four weeks later, four groups were fed a high fat diet (second hit; HFD32, CLEA, Japan) for 6, 8, 12, or 20 weeks. An additional control group was maintained on normal chow for 6 weeks. Afterwards, the animals were sacrificed and their livers were histologically processed. Sections of the left lobe were stained with H&E and scanned with a Hamamatsu NanoZoomer HT 2.0 whole-slide scanner at a resolution of 227 nm/pixel.

Steatosis scores were expected to vary significantly between groups because the animals were subjected to the dietary protocol for different periods of time. However, only insignificant differences were expected within groups because the respective animals were of the same strain and fed with the same diet for the same time.

Mouse livers were obtained from Stelic Institute & Co., Inc., Tokyo, Japan. Their transportation to Germany was approved by LANUV NRW, Recklinghausen, Germany.

#### Data set C

Data set C contained 30 whole-slide images showing serial sections of one mouse liver. The data set was divided into five groups of six consecutive sections each. The individual groups were about 300 μm apart, so that the five groups spanned a depth of about 1.2 mm. The consecutive sections were about 3 μm apart.

The animal was a male C57/BL6N mouse that was fed a methionine-choline-deficient high fat diet (Ssniff Spezialdiäten GmbH, Soest, Germany) for 4 weeks. Afterwards, it was sacrificed and its liver was histologically processed. Serial sections were cut from the center of the liver using a rotary microtome, stained with H&E, and scanned with a Hamamatsu NanoZoomer HT 2.0 whole-slide scanner at a resolution of 227 nm/pixel.

Even though the groups were only 300 μm apart, small but significant differences were expected between groups because of intra-liver heterogeneity [22]. However, only insignificant differences were expected within groups because consecutive sections show almost the same tissue.

The mouse was obtained from Charles River Laboratories, Sulzfeld, Germany. All procedures were carried out in accordance with German animal welfare legislation.

### Statistical analysis

We pursued a clinimetric approach to evaluate the considered scores, which is a common way to evaluate clinical measures [23, 24]. In the clinimetric approach, the ability to measure changes or differences in a clinical parameter is assessed in terms of reliability and validity. We used two descriptive statistics to quantify the reliability and validity of steatosis scores, the intraclass correlation coefficient (ICC) and the Kendall’s tau rank correlation coefficient.

#### Reliability

Reliability represents the extent to which distinct levels of the measured concept can be distinguished from each other, despite measurement errors [24]. Reliability is often used synonymously with precision and reproducibility. It is typically quantified by an intraclass correlation coefficient (ICC) that assumes values between 0 for poor and 1 for perfect reliability [25].

In our case, the ICC was computed as the ratio of the variance between groups to the total variance, that is, the variance between plus the variance within groups. The variance within groups was assumed to represent measurement errors and biological variability. ICC values were estimated from variance components of a one-way analysis of variance (ANOVA). One-way ANOVA was used because the groups in the data sets consisted of different, randomly-selected animals.

#### Validity

Validity is defined as the degree to which a measure actually measures what it is supposed to measure [23]. It is often used synonymously with accuracy. A measure can be reliable without being valid. It is most straightforward to evaluate the validity of a novel measure by comparison to an established gold standard [24]. However, because of their novelty, there was no gold standard for the focused scores presented in this paper.

When no gold standard is available, validity is commonly assessed through correlation with other features which are assumed to be related [24]. For data set A, we assumed that groups were sorted according to their steatosis levels. Therefore, we assessed the correlation between steatosis scores and diet indices. For data set B, we assumed that steatosis increased over time with continued feeding. Therefore, we assessed the correlation between steatosis scores and feeding time. For data set C, we could not make reasonable assumptions about the order of steatosis levels. Therefore, we refrained from evaluating validity on this data set.

Correlation was quantified using Kendall’s tau rank correlation coefficient [26], which does not depend on linearity and can account for ties in the data, such as multiple values per group. Kendall’s tau values range between −1 and +1, signifying negative and positive correlation, respectively. A value of 0 implies no correlation.

## Results

### Tile size evaluation

We evaluated focused scores based on different tile sizes in terms of their clinimetric quality and compared them against the standard score. Like the standard score, all considered focused scores were based on the mean. The evaluation was conducted on all three data sets. The resulting ICC and Kendall’s tau values are summarized in Table 1.

**Table 1:** Results of the tile size evaluation on data set A, B, and C. Kendall’s tau values were only computed for data set A and B because validity assumptions could only be made for these data sets. The tile size of the standard score was labeled as not applicable (N/A) because this score is practically unaffected by the tile size

Score	Tile size	Statistic	ICC A	ICC B	ICC C	tau A	tau B
standard	N/A	mean	0.86	0.54	0.14	0.78	0.60
focused	8 μm	mean	0.84	0.76	0.72	0.79	0.73
focused	16 μm	mean	0.94	0.83	0.79	0.81	0.76
focused	32 μm	mean	0.92	0.86	0.83	0.81	0.82
focused	64 μm	mean	0.87	0.77	0.67	0.78	0.79
focused	128 μm	mean	0.86	0.62	0.28	0.77	0.70

Focused scores always performed comparably to or better than the standard score. While all scores achieved excellent reliability and validity on data set A, focused scores performed substantially better than the standard score on data set B and data set C. However, the performance of focused scores strongly depended on the tile size. The best reliability was obtained with tile sizes of 16 μm or 32 μm. Of these two, a tile size of 32 μm achieved the best overall results on all data sets. The quality gradually dropped when the tile size became smaller or larger.

**Fig. 5:**
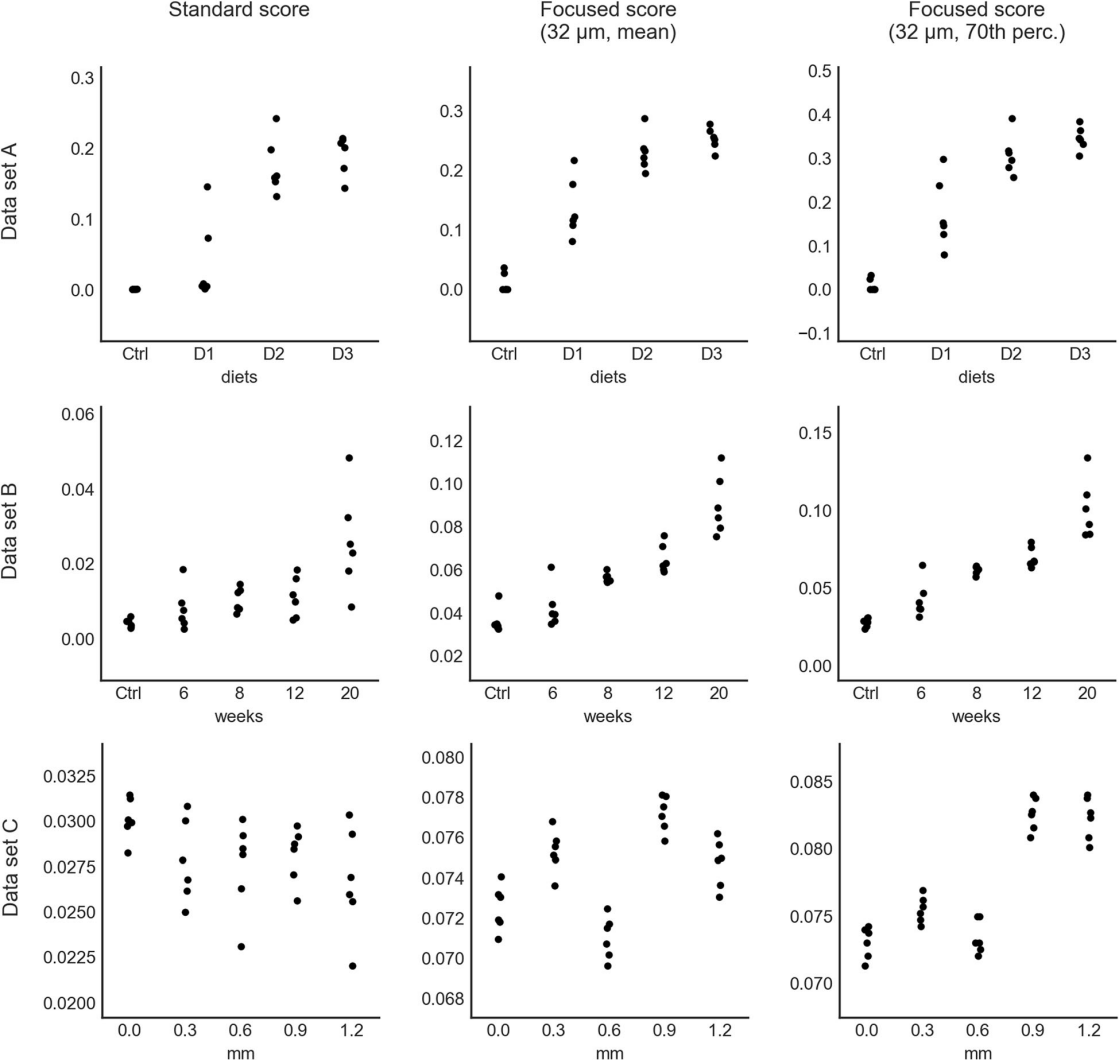
Score values. Distributions of score values obtained on data set A, B, and C. The horizontal axes give the groups of the respective data set, the vertical axes give the respective score values. Every dot represents one image. For better readability, the dots where randomly displaced by small amounts in horizontal direction

Figure 5 plots the values of the standard score and the focused score computed with a tile size 32 μm obtained on the three data sets. It becomes apparent that there is substantial overlap between the standard score values of the different groups. The focused score values, on the other hand, tend to be better separated between groups and more tightly clustered within groups.

### Percentile evaluation

We evaluated focused scores based on different percentiles in terms of their clinimetric quality and compared them against the mean-based focused score. The evaluation was conducted on all three data sets. Again, the best results were obtained with tile sizes of 16 and 32 μm. For brevity, we only present results obtained with a tile size of 32 μm because they consistently ranked among the best. The corresponding ICC and Kendall’s tau values are listed in Table 2.

**Table 2:** Results of the percentile evaluation on data set A, B, and C. Kendall’s tau values were only computed for data set A and B because validity assumptions could only be made for these data sets

Score	Tile size	Statistic	ICC A	ICC B	ICC C	tau A	tau B
focused	32 μm	mean	0.92	0.86	0.83	0.81	0.82
focused	32 μm	10th perc.	0.81	0.75	0.28	0.75	0.74
focused	32 μm	20th perc.	0.84	0.79	0.66	0.80	0.73
focused	32 μm	30th perc.	0.86	0.79	0.71	0.82	0.78
focused	32 μm	40th perc.	0.87	0.80	0.84	0.80	0.81
focused	32 μm	50th perc.	0.88	0.84	0.91	0.80	0.83
focused	32 μm	60th perc.	0.89	0.85	0.94	0.80	0.84
focused	32 μm	70th perc.	0.91	0.87	0.93	0.80	0.86
focused	32 μm	80th perc.	0.93	0.88	0.85	0.81	0.86
focused	32 μm	90th perc.	0.94	0.87	0.72	0.81	0.84

Focused scores based on high percentiles tended to perform better than focused scores based on low percentiles. This applied to all data sets and both the reliability and validity. However, on data set B and C, focused scores based on very high percentiles (80th, 90th) proved to be less reliable than focused scores based on somewhat smaller percentiles. The best overall results were achieved by the focused score based on the 70th percentile. While its performance was comparable to the mean-based focused score on data set A and B, its reliability was considerably higher on data set C.

The values of the 70th-percentile-based focused score are plotted in the third column of Fig. 5. Apart from being on a different scale, the value distributions of the percentile-based and mean-based focused scores were nearly indistinguishable on data set A. On data set B, there appears to be less intra-group variation in the percentile values and the 6 week group appears to be better separated from the control group. On data set C, the percentile values of the last two groups were much better separated from the values of the first three groups than the corresponding mean values.

## Discussion

Focused scores appear to be better suited for the quantification of steatosis in histological images than the standard score. As evidenced by their superior performance on data sets B and C, their particular advantage is the reliable discrimination of small differences in steatosis. By focusing only on steatotic tiles, the scores become insensitive to the sampling of non-steatotic tissue. Also, since focusing only on steatotic tiles increases their value, the scores are less sensitive to image analysis errors.

However, the performance of focused scores strongly depends on the tile size. If the tile size is too small, then most tiles lie either completely within or outside of macrovesicular fat droplets. In this case, scores are poorly resolved and, at the extreme, only assume the values zero or one. If the tile size is too large, then tiles cover substantial areas of non-steatotic tissue. In this case, focused scores become sensitive to the spatial heterogeneity of steatosis in the same way as the standard score. Interestingly, the best results were obtained when the tile size approximately matched the size of single hepatocytes (16 to 32 μm).

Focused scores based on percentiles can potentially further improve the discrimination of very small differences in steatosis over mean-based focused scores. This is suggested by the superior reliability of the focused score based on the 70th percentile on data set C. However, in the absence of validity assumptions for data set C, it is impossible to say whether the result is meaningful in practice. The superior performance of scores based on high percentiles over scores based on low percentiles can be explained with the premise of hotspot analysis, namely, that particularly high values are characteristic for the parameter distribution [15]. Very high percentiles, on the other hand, often capture inevitable artifacts, like vessels or cracks that were incorrectly classified as fat droplets. This might explain why the highest percentiles were less reliable than somewhat smaller percentiles on data set B and C.

Our results mirror findings obtained in the tile-based hot spot analysis of other histological parameters. Nawaz et al. also found the tile size to be a major factor in the prognostic quality of scores for estrogen-receptor-negative breast cancer [20]. Likewise, Plancoulaine et al. elaborate on the sensitivity of very high percentiles to artifacts in the context of the Ki67 proliferation index [19].

Computing focused scores is conceptually simple and straightforward to standardize, which is essential for widespread adoption and reproducibility. Also, it requires no manual interaction and virtually no additional computational costs over the standard score. The focused scores approach is complementary to many previously published methods for computing steatosis area fractions [9–14]. Deriving focused scores from these methods can be a simple way to improve their reliability. It must be pointed out, however, that the values of focused scores are on a different scale and, therefore, incomparable to the standard score.

Having reliable means for discriminating small differences in steatosis reduces the number of samples needed for demonstrating significant effects in research studies. This will not only reduce the effort of conducting studies, but in studies using animal models, it will also reduce the number of animal experiments required. Focused scores are likely to be beneficial in clinical practice as well, because the microscopic appearance of steatosis in human liver tissue is similar to the one in rodent tissue. However, further studies are necessary that evaluate how different states of fatty liver disease are reflected in different focused score values. Only when clinical guidelines are specifically tailored to focused scores, it will be possible to make full use of their increased reliability.

Focused scores were superior to the standard score on all three data sets used in the evaluation. Nevertheless, to prove their general superiority, they must be evaluated on further data sets. These should cover a broad range of applications and ideally also include human liver tissue. Performing a clinimetric evaluation for human tissue is much more difficult because of the impossibility to evaluate reliability in controlled experiments.

Besides steatosis, there are many histological parameters that are heterogeneously distributed across tissue sections. This includes scores quantifying the expression of biomarkers like Ki67, hormone receptors, HER2, or PD-1/PD-L1, which are important prognostic or predictive factors in the treatment of cancer. Future work should, therefore, be invested in evaluating whether focused scores can make the assessment of these biomarkers more reliable as well.

## Conclusion

Focused scores enable reliable quantification of heterogeneously distributed steatosis in histological images. They appear to be generally superior to the steatosis area fraction across the entire tissue, which is computed by most automated image analysis methods. Their superiority was particularly evident in the discrimination of small differences in steatosis. Focused scores are conceptually simple and straightforward to use in research studies. Provided that an appropriate tile size is used, their high reliability can potentially reduce the number of samples needed for demonstrating significant effects.

## Abbreviations

FLD: Fatty liver disease
H&E: Hematoxylin and Eosin
ICC: Intraclass correlation coefficient
